# Tobacco industry’s elaborate attempts to control a global track and trace system and fundamentally undermine the Illicit Trade Protocol

**DOI:** 10.1136/tobaccocontrol-2017-054191

**Published:** 2018-06-13

**Authors:** Anna B Gilmore, Allen W A Gallagher, Andy Rowell

**Affiliations:** 1 Tobacco Control Research Group, University of Bath, Bath, UK; 2 UK Centre of Tobacco and Alcohol Studies, University of Bath, Bath, UK

**Keywords:** illegal tobacco products, surveillance and monitoring, tobacco industry

## Abstract

**Background:**

The Illicit Trade Protocol (ITP) requires a global track and trace (T&T) system to reduce tobacco smuggling. Given the tobacco industry’s (TI) historical involvement in tobacco smuggling, it stipulates that T&T ‘shall not be performed by or delegated to the tobacco industry’. This paper explores the rationale for & nature of the TI’s effors to influence the ITP & its T&T system.

**Methods:**

Analysis of leaked TI documents and publicly available data, investigation of front groups, trademark and patent ownership.

**Findings:**

Growing & diverse sources of evidence indicate that the TI remains involved in tobacco smuggling and that TI cigarettes account for around two-thirds of the illicit cigarette market. The TI therefore has a vested interest in controlling the global T&T system aimed to curtail this behaviour. To this end, Philip Morris International (PMI) adapted its pack marker system, Codentify, to meet T&T requirements, licensed it for free to its three major competitors who then collectively promoted it to governments using front groups and third parties including companies claiming to be independent despite clear TI links. PMI also sought to suggest Codentify was independent by selling some parts of its intellectual property on Codentify while retaining others, leaving a complex web of shared interests. In Africa, British American Tobacco used payments to obtain data suggesting its smaller competitor companies were evading taxes and secure influence with tax authorities. Regulatory capture has been enhanced by a public relations effort involving TI funding for conferences, training, research, and international police and anti-corruption organisations. Collectively this has created public messaging and a powerful network of organisations supportive of the TI’s misleading postion on illicit.

**Conclusions:**

Governments should assume the TI seeks to control T&T systems in order to avoid scrutiny and minimise excise tax payments and that any T&T system based on Codentify, on intellectual property currently or previously owned by the TI, or being promoted or implemented by companies with TI links, is incompatible with the ITP and would not serve to reduce illicit trade.

## Introduction

The Framework Convention on Tobacco Control’s (FCTC) Illicit Trade Protocol (ITP), adopted in November 2012 following 4 years of negotiation^1^ (see Timeline in table 1), aims to eliminate all forms of illicit tobacco, but focuses particularly on securing the supply chain of legally manufactured tobacco products. A global track and trace (T&T) system which can track a tobacco product through its distribution chain and, should it enter the illicit market, ‘trace’ it back to determine at what point it entered the illicit channel is therefore central.^1^ This will be achieved by each party to the protocol requiring that every pack manufactured in or imported to their territory has a unique, secure marking providing information on manufacture, shipping and distribution. This focus and the stipulation that obligations for T&T systems ‘shall not be performed by or delegated to the tobacco industry’ were driven by overwhelming evidence of the transnational tobacco companies’ (TTCs) historical involvement in cigarette smuggling.^1–10^

**Table 1:** Timeline of events, 1998 to 2017

Timeline			
Date	Framework Convention on Tobacco Control (FCTC) and Illicit Trade Protocol (ITP)	European Union (EU)	Tobacco industry (TI)
1998		EU investigation of transnational tobacco company (TTC) cigarette smuggling starts^16^	
December 1998			An affiliate of RJ Reynolds pleads guilty in US tobacco smuggling lawsuit and is fined $15 million^170^
June 1999			RJR MacDonald’s parent company under investigation by the Royal Canadian Mounted Police (RCMP) for complicity in tobacco smuggling between 1989 and 1994^171^
December 1999			Through US Courts, the Canadian government sues RJ Reynolds and affiliates in alleging they were part of a conspiracy to smuggle cigarettes into Canada^12^
June 2000			Canada’s lawsuit rejected on the grounds that US courts cannot be used to collect the taxes of another country^172^
November 2000		EU files Racketeer Infuenced & Corrupt Organizations Act (RICO) case in New York Court against TTCs accusing the companies of ‘an ongoing global scheme to smuggle cigarettes’^16 173 174^	
August 2001		10 EU member states join the lawsuit^174^	
January 2002		Additional charges filed against Japan Tobacco International (JTI) and its affiliates^175^	
October 2002		Additional allegations filed against RJR MacDonald 16	
February 2003			The RCMP file criminal charges against RJ Reynolds and affiliates over cigarette smuggling in the 1990s^14^
May 2003	The FCTC is adopted by the World Health Assembly^176^		
July 2004		EU and member states drop case against Phillip Morris International (PMI) in return for enforceable and legally binding agreement. PMI pays the EC $1250 million over 12 years.^16^ Through this and subsequent agreements with the other TTCs, they collectively had to make payments of US$1.9 billion to the EU and its member states, additional payments if their tobacco products were, through seizures, found on the illicit market (although only in large numbers)^16^ and to mark their products with trackable codes^177–180^	
October 2004			PMI files priority international patent for ‘methods and systems for marking, tracking and authentication of products’ (Codentify)^77^
February 2005	The FCTC enters into force^181^		
September 2005			International Codentify patent filed^77^
February 2006	Conference of the Parties (COP) 1—officers elected and main committees established^182^		
September 2006			Codentify patent enters the European regional phase^183^
June 2007	COP 2—decision to establish an intergovernmental negotiating body (INB) on the ITP^182^		
December 2007		EU reaches agreement on illicit trade with JTI. JTI agrees to pay the EC $400 million over 15 years^179^	
February 2008	INB—first meeting (negotiations for ITP begin)^182^		During 2008–September 2010, the four major tobacco companies in Canada plead guilty to tobacco smuggling and were collectively fined $C1.7 billion^14^
October 2008	INB, second meeting^182^		
November 2008	COP 3^182^		
February 2009			Codentify patent is granted by Eurasian Patent Organization^184^
April 2009			Codentify patent is granted in Europe^185^
June 2009	INB, third meeting^182^		
March 2010	INB, fourth meeting^182^		
July 2010		EU reaches agreement on illicit trade with British American Tobacco (BAT). BAT agrees to pay the EC $200 million over 20 years^177^	
September 2010		EU reaches agreement on illicit trade with Imperial Tobacco Limited. Imperial agrees to pay the EC $300 over 20 years^178^	
November 2010	COP 4—decision to establish informal working group on the ITP^182^		PMI licences Codentify for free to its main competitors.^65^Tobacco Industry Working Group on Digital Tax Verification formed^67^
May 2011			The first Project Star report published on illicit tobacco in the EU, commissioned by PMI from KPMG^25^
July 2011	ITP working group holds its first meeting^186^		
October 2011			Digital Coding and Tracking Association (DCTA) registered in Zurich^187^
March 2012	INB, fifth meeting^182^		
June 2012			PMI makes €15 million donation to Interpol to work with DCTA^132^
November 2012	COP 5—ITP adopted and Interpol (in receipt of pounds from PMI) applies for observer status^182 188^		
December 2012			SICPA awarded Kenyan Revenue Authority tender, despite lobbying efforts for Codentify by BAT and FractureCode^189^
January 2013	ITP opened for signature^190^		
April 2013			European Codentify patent is updated to change the applicant from PMI to the DCTA^191^
September 2013			PMI donates €55 000 to International Anti-Corruption Academy (initiated by European Antifraud Office and UN Office on Drugs and Crime)^131^
December 2013	EU signs ITP^192^		US Codentify patent is filed^193^
April 2014		The Revised Tobacco Products Directive (TPD) agreed. Articles 15 and16 relate to T&T and operationalise the ITP in the EU^194^	DCTA is the major sponsor of World Customs Organization conference on illicit tobacco^6^
May 2014			KPMG & GS1 UK release a DCTA-funded report promoting Codentify^138^
June 2014			First Project SUN report is published—a continuation of Project Star but now commissioned by all TTCs^195^
October 2014	COP 6—Report on the status of the ITP. Request for establishment of ITP expert panel^182^		
November 2014			BAT is fined £650 000 (later reduced to £10 000)^35^ for oversupplying products to Belgium^34^
March 2015		Feasibility assessment on EU T&T system published^117^	
June 2015			Coalition Against Illicit Trade is formed^118^
April 2016			Inexto established^101^
May 2016	Coordinating meeting of the ITP expert panel^196^		PMI launches PMI IMPACT^197^
June 2016	EU ratifies ITP^198^		DCTA announces that it has sold Codentify to Inexto, and PMI claims it now complies with the TPD^199^ PMI IMPACT announces first call for proposals to fund^160^
		Inception impact assessment for delegated acts under Articles 15 and 16 of TPD published^200^	
July 2016		EU agreement on illicit trade with PMI expires^201^ EU public consultation on EU system of T&T in line with Articles 15 and 16 of the TPD (ends in November 2016)^202^	
September 2016			DCTA transfers ownership of Codentify’s European patent to Inexto^203^
November 2016	COP 7—Parties urged not to consider tobacco industry proposals or assistance on T&T. Requests ITP expert panel to report at next COP^182^		Two trademarks for Codentify, covering Switzerland and the EU member states, are transferred to Inexto^112–114^;
June 2017	First meeting of ITP expert panel^196^		
September 2017		Consultation on draft implementing regulation on technical standards for T&T system^168^	PMI IMPACT funds 32 projects and many led by organisations with previous TI links^159^

This paper aims to examine the nature and purpose of TTC efforts to undermine the ITP and the implications for global tobacco control. Through analysing data on the structure of the illicit tobacco market, leaked industry documents, patent and trademark filings and investigating front groups, it shows that TTCs are engaged in an elaborate campaign to control the global T&T system the ITP envisages by promoting its own pack marker system, Codentify (box 1), as the T&T system of choice.

Box 1 **Codentify versus enhanced tax stamp systems**

Codentify: A code-generating system developed and promoted by the transnational tobacco companies (TTCs). Initially developed as a non-secure authentication system (to determine if a product is authentic or counterfeit), it was subsequently adapted for use as a digital tax verification system.^65 204^ Installed at the production line, the system prints two unique codes on each tobacco/cigarette packet—a production information code detailing, inter alia, line and time of production, and a 12-character alphanumeric code generated through an encrypted digital signature to the production information code.^66 193^ There is no linked security feature. Tobacco industry insiders, academics and the Framework Convention on Tobacco Control’s Secretariat have criticised Codentify as an inefficient^205^ and ineffective track and trace (T&T) mechanism.^79 206^

Enhanced stamp systems: Developed initially to focus on individual packs (not cartons, master cases or pallets) intended for the domestic market and to enable volume reporting and revenue collection, tax stamps have now been advanced through the addition of enhanced security features and database linkage to allow T&T and authentication of genuine versus counterfeit products. The key feature is the combination of digital (the unique identification code on a pack) and physical security elements (these may be overt, eg, holograms; covert, eg, fluorescent fibres; or forensic) which make new tax stamps difficult to counterfeit.^167^

Codentify-based system has close links to the tobacco industry, while tax stamp systems were developed independently. Tax stamp producers, also in the business of printing secure documents for government (passports, ID documents, currency), are subject to international standards that control their production and distribution processes.

## Evidence of historical and ongoing tobacco industry involvement in tobacco smuggling

### Pre-1990

TTCs have a long history of complicity in tobacco smuggling.^2 4–10^ They profit when they sell to the distributor regardless of whether their product then enters the illegal market.^4^ Tobacco smuggling can benefit them in numerous ways (box 2).

Box 2 **The ways in which tobacco smuggling can benefit transnational tobacco companies (TTCs)**

- Smuggled tobacco has either no excise duties or duties from a lower tax jurisdiction applied. Consequently, it is sold for less than it should be. The cheaper a product, the more it sells, especially to the most price-sensitive smokers—the young and the least well off.^6^
- Smuggling undermines tobacco control measures making them less effective in reducing smoking. An obvious example is tobacco taxes, but because illicit product is not usually sold through standard outlets, it also undermines age of sale controls and licensing.^6 17 207^
- Smuggling is a key market entry technique that the TTCs have used extensively^208^ to bypass tariff and non-tariff barriers to trade and move tobacco into closed or protected markets. Simultaneously, TTCs argue that the presence of illicit products signals a need for them to invest in that market (rather than resulting from their involvement in the illegal trade).^9 208^
- TTCs use tobacco smuggling to oppose tobacco control policies, arguing that demand for the illicit product, rather than its supply, drives the problem and the tobacco control policy in question will only make this worse. Historically TTCs mainly applied this argument to tobacco taxes,^24^ often causing countries to reduce their tobacco excise rates.^4 207^ The problem of tobacco smuggling is now used to oppose almost every tobacco control policy.^19 24 209^

*Source*: Adapted from Gilmore *et al.* 6

Through the 1990s, overwhelming evidence from TTC documents detailed their involvement in global cigarette smuggling.^2–10^ The scale was unprecedented—a third of global cigarette exports were estimated to end up on the illicit market^4^ with TTCs supplying some markets almost entirely via illicit channels.^9 10^By the late 1990s, investigations and lawsuits (table 1)^11–14^ had led to guilty verdicts^14 15^ and legal agreements including between the European Union (EU) and all four TTCs—Philip Morris International (PMI), British American Tobacco (BAT), Japan Tobacco International (JTI) and Imperial Tobacco.^16^

### Post-1990: a change in the competitive landscape for illicit tobacco products

With their activities exposed, TTCs changed their export practices.^17^ Total illicit cigarette volumes declined,^17^ but new types of illicit products began to appear alongside tobacco industry illicit—counterfeits and cheap whites (box 3).^17 18^ Simultaneously, TTCs sought to shift the issue from a public relations (PR) disaster where they were the pariah supplier of illicit product^8^ to a PR success story identifying them as both the victim of and solution to tobacco smuggling.^6^ They did so by using their resource advantage to purchase data, access and influence,^6^ exaggerate the threat of illicit tobacco (particularly counterfeit and cheap whites) and present them as a consequence of tobacco control policies.^19–22^

Box 3 **Types of illicit tobacco products now being seen**

**Counterfeits**

- Products bearing a trademark of a cigarette manufacturer that are manufactured by a third party without consent from that cigarette manufacturer.

**Cheap whites (also known as illicit whites)**

- Non-transnational tobacco company (TTC)-branded cigarettes that are legally produced but have no legitimate market. This confusing term initially used by TTCs is defined by the European Commission as: ‘brands manufactured legitimately in one market, either taxed for local consumption or untaxed for export, and sold knowingly to traders who transport them to another country where the products are sold illegally without domestic duty paid.’^210^

**Tobacco industry illicit (tobacco industry product present in the illicit market)**

- Product of one of the cigarette manufacturers that was en route to, imported into, distributed in or sold in a jurisdiction in violation of that jurisdiction’s fiscal laws. That this product was manufactured by a tobacco company does not imply the company is always responsible when that product ends up on the illicit market.

Source: Adapted from Gilmore *et al*.^25^

### Ongoing industry involvement: emerging evidence and data

Recent data consistently show that at global, European and national level, the majority of the illicit cigarette market still comprises tobacco industry product (table 2). Latest estimates suggest that approximately 60%–70% of the illicit market is tobacco industry product with specific figures varying from 58% (2016, EU level, industry funded data) to 69%–73% (seizure data for 2011 and 2012 at global level and 2014 and 2016 at UK level). This has occurred despite the use of Codentify in, according to industry claims, over 100 countries worldwide (online supplementary appendix 1).^23^

10.1136/tobaccocontrol-2017-054191.supp1 Supplementary file 1

**Table 2:** The make-up of illicit cigarette market by type (tobacco industry illicit, cheap whites and counterfeit): recently available data at global, European and UK levels

	2007	2008	2009	2010	2011	2012	2013	2014	2015	2016*
Global (WCO data)										
Illicit white					20%	25%	NA	NA	NA	
Counterfeit					7%	7%	4%	2%	2%	
TI illicit					73%	69%	NA	NA	NA	
EU (Project Star and Project Sun)										
Illicit white	4%	8%	13%	15%	23%	26%	33%	37%	35%	34%
Counterfeit†	6%	6%	5%	5%	4%	0%	6%	7%	9%	8%
TI illicit	89%	86%	82%	80%	74%	74%	61%	56%	56%	58%
UK (Operation Henry 1&2)										
Illicit whites								24%		14%
Counterfeit								5%		18%
TI illicit‡								72%		69%

Blank cells are where no reports were published. NA indicates where a report was published but a specific data item was not available.

WCO data taken from the WCO Illicit Trade Reports: 2014 and 2015 data from 2015 report^211^; 2013 data from 2014 report^212^; 2012 and 2011 data from 2012 report (the first such report)^213^ (please note figures differ very slightly between reports) (based on seizure data).

EU data taken from the Project Sun and Project Star reports published by KPMG and funded by the tobacco industry and the Royal United Services Institute^214–220^ (based on industry data and modelling by KPMG).

UK data taken from the Operation Henry reports published by the Chartered Trading Standards Institute and commissioned by the Department of Health Tobacco Policy Team^27 221^ (based on systematically collected seizure data).

*2016 Operation Henry data were collected from December 2015 to April 2016 inclusive.

†The Counterfeit data in the Project Sun/Star reports comprise just counterfeit PMI brands from 2007 to 2011 and counterfeited brands for all four TTCs from 2013 onward.

‡The 2016 Operation Henry report listed the two most seized products, West and Winston which are tobacco industry brands (sold in the UK by Imperial Tobacco and Japan Tobacco International, respectively), as cheap white products. In our analysis, these have instead been included as TI illicits. This is more likely to give an accurate picture because, although it is unclear if a determination was made as to whether these products were genuine or counterfeit, as they are not widely sold in the UK it is thought unlikely that counterfeiters would target them at the UK market.

EU, European Union; PMI, Philip Morris International; TI, tobacco industry; TTCs, transnational tobacco companies; WCO, World Customs Organization.

By comparison, the problem of counterfeit, which the industry continuously emphasises,^6 19 20 24 25^ comprises only 5%–8% of the illicit market (other than in the 2016 *Operation Henry* data which are problematic—see footnote to Table 2). The contribution of cheap whites represents, in most of these data, around a fifth to a third of the illicit market. There has, however, been some confusion in defining and measuring cheap whites. For example, industry-commissioned *Project Star* report, undertaken by KPMG, incorrectly classified the Imperial Tobacco brand, Classic, as a cheap white during a period (2006–2012) when it was one of the most seized brands in Europe.^26^ Similarly, in the latest *Operation Henry* report, the two most seized brands, West and Winston, were coded as cheap whites yet are TTC brands.^27^ Consequently, data may underestimate tobacco industry illicit.

While the smuggling of some tobacco industry cigarettes may be outside their control, the sheer volume suggests some involvement. Whistleblowers,^28^ researchers,^16^ investigative journalists,^29 30^ alongside government reports,^31 32^ investigations,^33^ accusations^33^ and fines^34 35^ suggest that industry involvement has continued since the 1990s. At best, evidence indicates that tobacco companies are failing to control their supply chain, overproducing in some markets (eg, Ukraine^29^) and oversupplying to others (eg, Belgium) in the knowledge their products will end up on the illicit market. At worst, ex-employees insist JTI remained actively involved, describing ‘rampant smuggling’ throughout the Middle East, Russia, Moldova and the Balkans.^28^ Leaked documents suggest that BAT staff suspected JTI was facilitating smuggling into the Democratic Republic of Congo (DRC)^36 37^ but that BAT also clandestinely moved millions of dollars in cash from Uganda to the DRC to buy tobacco leaf which was presumably then illegally exported.^38 39^ In 2011–2012, BAT cigarettes being distributed by a company previously implicated in tobacco smuggling were ending up in the illicit market across Africa, the Middle East and Europe with BAT staff agreeing not to discuss the problem by email.^40–42^

Evidence suggests that smaller tobacco companies in Africa are also involved in smuggling and that, despite evidence of its own involvement,^33 39–42^ BAT sought to prove these companies were evading tax payments, using this knowledge to undermine them and gain influence with tax authorities.^30 33 43–57^ In South Africa, critics claim BAT engaged in money laundering to fund a large spy network and used its funding and the data obtained to secure a seat on the multiagency Illicit-Tobacco Task Team where it could then drive the law enforcement agenda.^33 43 44 48 57^ While such detailed evidence is limited to South Africa, BAT documents indicate the company was also paying informants to obtain competitor data elsewhere in Africa and using these data, alongside payments to staff,^58 59^ to ingratiate itself with tax authorities.^60–64^ Collectively this evidence suggests a very real danger of regulatory capture.

### Implications for T&T

It is unsurprising that tobacco industry illicit has not fallen further given that the incentives (box 2) have hardly changed and, where evaluated, fines are too small to offer sufficient deterrent to ongoing involvement.^16 25^ At EU level, for example, seizure payments paid by the TTCs from 2006 to 2012 cover only 0.08% of estimated government excise losses despite TTC product representing at least 74% of illicit tobacco over that period (table 2).^16 25^

Effective and well-implemented T&T systems run independently of the tobacco industry would make ongoing TTC involvement in illicit almost impossible. And, as they can only be applied to legally manufactured product, would disadvantage TTCs compared with operators selling counterfeit and cheap whites, providing an incentive for TTCs to control them.

## Tobacco industry interests in and influence on T&T systems: evidence from leaked industry documents and linked investigations

### Tobacco industry’s fears and aims

Leaked industry documents highlight TTCs’ fears and aims around illicit, tax stamps, T&T systems and the ITP.^65–69^ In 2003, BAT outlined how the industry was perceived as ‘part of the problem’ in illicit yet needed ‘to be part of solution to combat threat to our business’.^68^ It was identified as ‘VITAL for Big Tobacco to be involved in shaping final regulation’ in this area.^68^ To this end ‘cooperation with Governments and Customs authorities worldwide’ was key.^68^ A later BAT document outlining the ‘building blocks’ of an Anti-Illicit Trade Advocacy strategy stressed the need ‘To reinforce British American Tobacco as being part of solution, not part of the problem’.^70^

Documents suggest TTCs feared the implementation of enhanced tax stamp systems such as those of a leading company in the field, SICPA,^65 66^ most notably the cost and lack of TTC control.^65 66^ The TTCs’ strategy appeared to involve three key elements: to collectively develop their own alternative, Codentify (box 1), and promote it to governments as a digital tax verification (DTV) and T&T system^65 66 71^; to actively oppose tax stamp systems and convince governments they were inferior to Codentify^65 66 71^; and to ‘proactively shape T&T regulation’^66^ to enable the above.^66^ BAT Whistleblower Paul Hopkins’ Employment Tribunal documents allege that he was tasked by the company’s lead for Anti-Illicit Trade ‘to disrupt and if possible stop other service providers of DTV and T&T products from winning tenders …[because]…. BAT had developed its own preferred system in conjunction with Philip Morris International called Codentify and wanted this system to be adopted by as many countries as possible’.^72^

Engaging governments and tax authorities in order to promote common standards on T&T that would help secure the implementation of Codentify over tax stamps appear to have been key.^65 71^ Documents note, for example, that: ‘Manufacturers should be involved in providing advice and assistance on best practice solutions to governments intending to institute new systems and, where appropriate, should participate in the drafting process, for example in relation to any proposed Framework Convention on Tobacco Control (FCTC) Illicit Trade Protocol’.^69^

The TTCs closely monitored ITP negotiations^73^ and, despite being formally excluded, BAT was, at different stages, able to obtain confidential information^74 75^ apparently including the text of the protocol.^76^ A 45-page document setting out BAT’s campaign plan for the fifth Conference of the Parties in November 2012, where the ITP was adopted, noted BAT’s preferred outcome on T&T as ‘Stamping and coding should be digital (Codentify).’^71^

### Developing Codentify as a pan-industry product

Codentify was originally patented by PMI in the mid-2000s following its legal agreement with the EU^77^ (see Timeline in table 1). In late 2010, 2 years after ITP negotiations had begun, PMI licensed Codentify for free to the other TTCs who collectively established a Working Group to collaborate on ‘DTV’,^65 67^ promoting Codentify to governments as an alternative to tax stamps.^67^

### Promoting Codentify via an increasingly elaborate set of front groups

In line with the ITP specification that T&T systems cannot be ‘delegated to the tobacco industry’,^78^ the pan-industry agreement and linked documentation stipulated the importance of making Codentify appear independent.^65 67^ This need was later underscored when BAT noted that the South African Department of Health ‘voiced its concern and will not support an “Industry” solution.’^66^ The TTCs therefore began giving the impression of independence via a complex system of front groups and third parties.

#### Digital Coding and Tracking Association

The first of these front groups, the Digital Coding and Tracking Association (DCTA), was created by the TTCs in 2011 to promote Codentify to governments,^67 79^ a role it continues to perform.^80 81^ DCTA’s glossy brochure claimed Codentify could ‘meet the expected licensing provisions of [ITP] Article 5’ and deliver ‘Full Government control’ but failed to acknowledge it was developed and patented by the tobacco industry.^82^ It has promoted Codentify in a recent consultation, again failing to acknowledge industry links.^83^

#### FractureCode and ATOS

The pan-industry agreement also outlined the role for ‘an independent reputable organisation’ to promote Codentify:

When discussing DTV with authorities, it is important to stress that while the solution is developed and supported by the major industry players, the operation and control of the system will be handled by an independent reputable organization assigned by the respective government.^65 67^

Documentation outlines that this was necessary because governments ‘need to be convinced for themselves that this [Codentify] is a high quality solution, which works totally under their control and supervision, and which is supplied to them by a credible third party technology company.’^65^

Yet simultaneously it suggests that TTCs (rather than governments) would select these ‘independent’ organisations and had already pre-selected two—FractureCode and Siemens.^65^ Their role was to: ‘guarantee to governments that the “Codentify” system works’; ‘promote and sell the system to governments’; and ‘after winning a government tender… install the system’.^65^A later (2012) BAT email indicates that it was working ‘globally with two approved suppliers to represent Codentify,’ this time naming FractureCode and ATOS,^84^ both of which subsequently appear to have been involved in tendering for T&T systems on the TTCs’ behalf (see below). We identified no further evidence of Siemens fulfilling this role. However, Siemens is a long-standing supplier of tobacco manufacturing machinery, produces its own code reading systems^85^ and was reported to be involved in operationalising a T&T system for BAT in Poland, providing both hardware (a code reading system) and software.^86^ Moreover, ATOS was involved in developing Codentify^87 88^ and in December 2010 acquired Siemens’ IT services division for €850 million.^89^

FractureCode, a Danish company established in 2002, offers T&T, digital authentication and volume verification solutions including Codentify.^90^ Its web page claimed that Codentify is ‘Aligned with expected requirements of WHO FCTC Protocol on Illicit Trade in Tobacco Products’.^91^ Although the industry’s exact relationship with FractureCode was unclear even to BAT staff,^84 92^ interactions during a tender process in Kenya suggest a close relationship with and degree of control by BAT (box 4). BAT Whistleblower Paul Hopkins’ Employment Tribunal documents state that by 2011 FractureCode was ‘in the pay’ of BAT.^72^ As the first T&T system to be implemented in Africa post-ITP, Kenya’s tender outcome would have significant ramifications, making BAT fearful that SICPA’s product would be approved.^93^ Documents suggest that FractureCode was also representing Codentify in Mauritius, Uganda and possibly Germany.^84 92^

Box 4 **Links between British American Tobacco (BAT) and FractureCode illustrated by the tender process for a track and trace (T&T) system in Kenya**

In 2012, Kenya held a tender for tobacco revenue stamps with T&T and integrated product accounting systems. BAT did not tender for the service directly but instead used FractureCode to promote Codentify. As Eric Jones, BAT’s International Solutions Engagement Manager for Global Supply Chain Tracking and Verification, noted: “following the launch by the KRA [Kenyan Revenue Authority] of the tender that clearly favoured SICPA, we agreed the use of FractureCode (FCC) to support you [BAT Kenya] in fighting/amending/cancelling this tender.” He added: “It is worth noting that not using a third party such as FCC to respond to the tender is likely to severely reduce our ability to shape events and prevent SICPA from winning”.^93^

Other emails note that BAT had ‘purchased’ the tender on FractureCode’s behalf,^92^‘organised a consultant to represent them [FractureCode]’ at a KRA question and answer session and drafted a letter on FractureCode’s behalf.^92^ It appears this letter was to be sent by FractureCode to the Commissioner General of the KRA, saying: “We, FractureCode Corporation/Codentify, a well established Security Company in Denmark, promoting and selling Digital Tax Verification for Tobacco and Alcohol Products, would like to formally protest about the conduct of the recent KRA tender carried out by your authority.” 222 Other documents suggest BAT wrote the original draft.^223^

BAT also required FractureCode to ‘Work with the Danish Embassy/Foreign Affairs to get tender cancelled. (This borne by FCC).’^84^ Documents indicate that the Danish Embassy wrote a letter on FractureCode’s behalf and met with the KRA to help get the tender extended.^224^ On 4 May 2012, a Danish Embassy staff member in Nairobi emailed the minutes of their meeting with the KRA to FractureCode stating: “We believe the result of this meeting leaves room for your company to submit your bid and have a direct dialogue with the KRA throughout the process. The Embassy would be happy to assist you in facilitating the contract.”^225^ The Embassy sent FractureCode an invoice for 9 hours work at Kr915 (Danish Kroner) an hour.^225^ It is not known whether the KRA and Embassy understood the BAT link.

Despite these efforts, at the end of 2012, KRA awarded the tender to SICPA.^189^ The subsequent implementation of the T&T system was completed by March 2014 and government figures indicate a 49% increase in legal cigarette and cigar sales and a 20% increase in tobacco tax revenue from 2013 to 2015.^167^

French company, ATOS, originally involved in Codentify’s development^87 88^ and named in leaked BAT documents^84^ may have played a similar role to FractureCode. It has promoted Codentify in Asia^94^ and been involved in the implementation of Codentify in Lithuania alongisde DCTA.^95–98^

#### Inexto

The outing of DCTA as a tobacco industry front group in 2012^17^ limited the TTCs’ ability to argue that Codentify was independent. With efforts to operationalise the ITP accelerating, this was becoming increasingly important.^17^

On 24 June 2016, the EU became the 19th party to ratify the ITP.^99^ Three weeks before, DCTA announced it had sold Codentify to a company called Inexto, an affiliate of the French Group Impala,^100 101^ reportedly for only 1 Swiss Frank.^102–104^ Inexto had been established just a few weeks previously^101^ and when the handover was reported in the press, a PMI spokesperson claimed Codentify “now complies with … the WHO’s Framework Convention on Tobacco Control.”^105^

**Figure 1:**
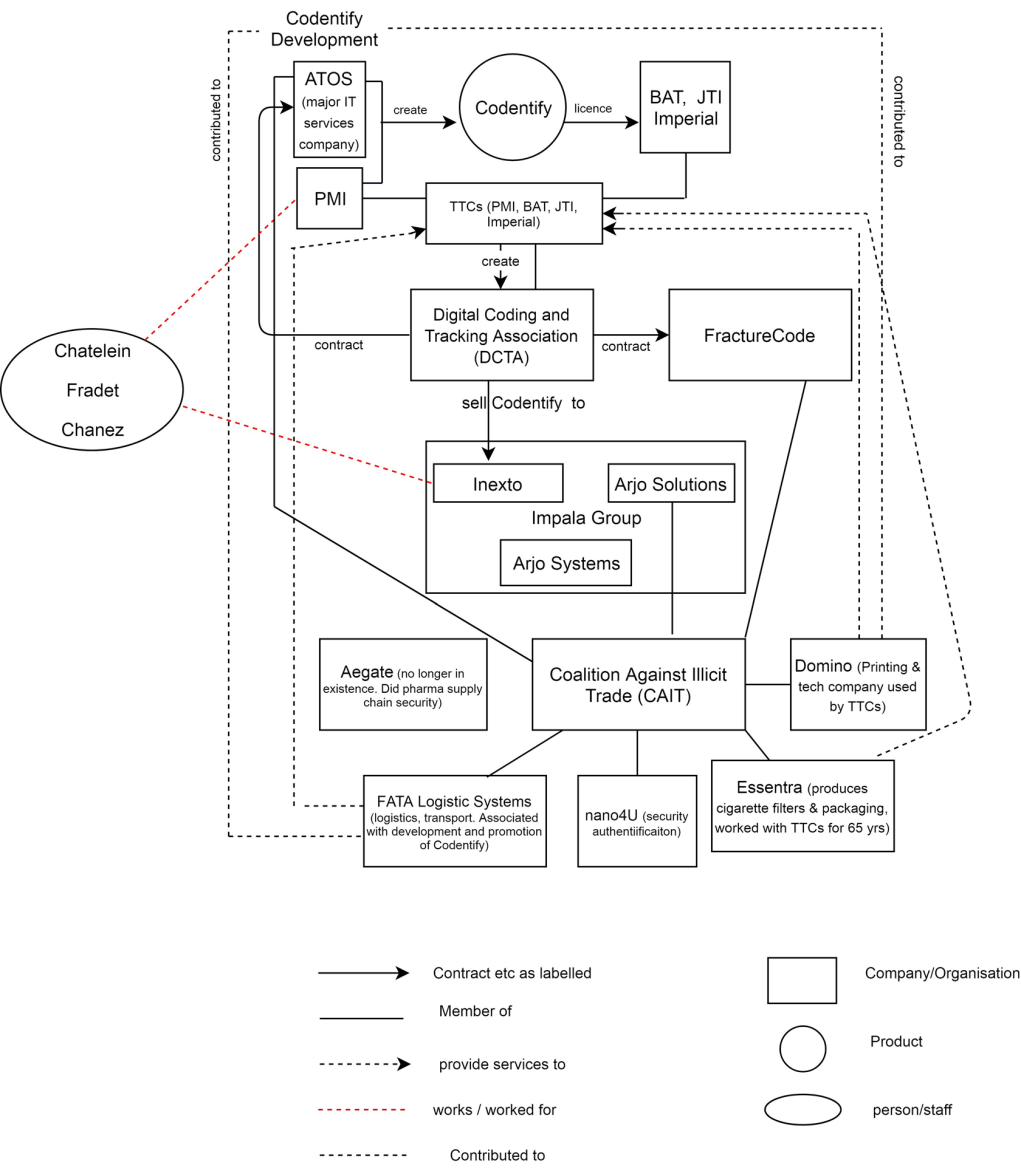
Diagram showing relationships between the creators and promoter of Codentify and the TTCs. BAT, British American Tobacco; JTI, Japan Tobacco International; PMI, Philip Morris International; TTCs, transnational tobacco companies.

Yet Inexto’s links to PMI are clear. Its managing director is Philippe Chatelain, previously PMI’s Director of Product Tracking Intelligence & Security for 14 years.^105 106^ Other top officials are Erwan Fradet, PMI’s Product Manager for Codentify for 5½ years,^107^ and Patrick Chanez, who worked for PMI for over 10 years developing anti-illicit trade technology.^108^ All three are coinventors of Codentify and still (as of November 2017) hold numerous patents with various PMI companies although now also hold some with Inexto and one with DCTA (Espacenet search 13 November 2017). Four months after the June 2016 announcement, Philip Morris Products SA still owned the global trademark rights to Codentify (online supplementary appendix 2, figure 1a) and Chatelain still held signing authority for that company (this ended 3 November 2016).^109^

10.1136/tobaccocontrol-2017-054191.supp2 Supplementary file 2

Following public criticism of the close link between PMI and Inexto,^105 110 111^ two of the trademarks for Codentify, covering Switzerland and the EU member states (online supplementary appendix 2, figure 1b), were transferred to Inexto in late November 2016.^112–114^ Chatelain has since suggested that the Codentify system has been redeveloped from scratch, again publicly implying it would be compliant with the ITP.^81^ Yet, an additional 20 Codentify trademarks in the WIPO database are still listed as being held by PMI companies covering, for example, Chile, USA, Indonesia, Israel, Mexico, Malaysia, Jordan and UAE, some with application dates as recent as March 2017.^115^Nevertheless, in June 2018 PMI stated to the Guardian newspaper: “We confirm that the worldwide assignment of all Codentify trademarks, previously owned by Philip Morris Products SA, to Inexto SA (part of Impala Security Solutions B.V.) was completed. It is up to Inexto SA to take steps to record the change of ownership at all relevant trademark registries, including WIPO.”

### The Coalition Against Illicit Trade (CAIT)

The latest group promoting ‘an industry operated solution’^116^ is the CAIT (figure 1), formed in June 2015 (3 months after the EU T&T feasibility assessment was published)^117^ and described as ‘a new worldwide coalition of businesses and organisations dedicated to fighting the trade of counterfeited and contraband goods’.^118^ As of November 2017, six of the seven members (an eighth, Aegate, has gone into administration^119^) can be linked to the tobacco industry. Yet its submissions to the EU T&T consultation^116^ and EU transparency register^120^ fail to mention tobacco industry links.

ATOS and FractureCode (see previous section) are members^121^ as is Inexto’s sister company within the Impala Group, Arjo Solutions.^121 122^ Of the other three, FATA Logistics was associated with the development and promotion of Codentify.^123^ Domino, a printing and technology company,^124^ claims to have worked closely with the Codentify development team and the tobacco industry for over a decade.^125^ Describing itself as a global provider of Codentify^126^ and the ‘tobacco industry’s coding technology supplier of choice’^127^ it is involved in a project to adapt Codentify to pharmaceuticals.^128^ Essentra, which produces cigarette filters and packaging (including security solutions such as holographic products and specialist inks), has been working with the tobacco industry for 65 years.^129 130^

### Engaging regulatory agencies and enhancing public relations efforts

A 2012 BAT presentation identified ‘key influencer stakeholder groups’ as central to ‘proactively shap[ing]Track and Trace regulation’. Listed stakeholders included the World Customs Organization (WCO), International Monetary Fund, Interpol and Organisation for Economic Cooperation and Development (OECD) alongside ‘Key influencer’ governments.^66^ Evidence suggests such efforts have been extensively operationalised creating a powerful network that promotes the TTCs’ position on illicit.

In 2011, PMI donated €55 000 to the *International Anti-Corruption Academy*, an organisation initiated by the European Antifraud Office (OLAF) and *UN Office on Drugs and Crime (UNODC)* to provide anti-corruption education and research.^131^

In 2012, PMI donated €15 million to *Interpol*, the world’s largest police organisation, to work with DCTA to promote Codentify.^79^ It made Codentify accessible to law enforcement agencies via Interpol’s Global Register^79 132 133^ and Interpol’s then Secretary General publicly promoted it.^134^ BAT’s Eric Jones outlined the importance of Interpol’s involvement stating it “will reinforce the credibility of the DCTA (and BAT) when talking to Governments as a credible provider of technology on DTV and T&T.”^135^

BAT flagged cooperation with WCO as important because of the ‘need for cross border/regional solutions shaped by WCO not WHO.’^68^ In 2014, the industry’s DCTA was a major sponsor of the WCO conference on illicit tobacco in Brisbane, Australia. KPMG’s Robin Cartwright presented in DCTA’s time slot and, despite then taking £10 million a year from PMI,^6 136^ failed to acknowledge this in his slides.^137^ Simultaneously, KPMG and GS1 UK launched a new report promoting Codentify^138^ which mentioned DCTA funding but not DCTA’s tobacco industry status.^6^ WCO also works closely with Interpol^139^ and other tobacco industry-linked groups, including the International Tax and Investment Centre,^140 141^ with whom WCO has cohosted conferences and training on tobacco smuggling.^142–144^

The *International Chamber of Commerce* has close links to and has repeatedly supported the tobacco industry.^140^ All four TTCs are members of its *Business Action to Stop Counterfeiting and Piracy* (BASCAP) initiative which purports to ‘combat product counterfeiting’.^140 145^ Through events which BASCAP organises or participates in, including an August 2016 UN Counter-Terrorism Centre meeting, TTCs are given a platform to present their position on illicit trade, counterfeiting and crime.^140 146–149^

In addition to these international efforts, TTCs have been working with national Governments^3^ and via ex-policemen and front groups established by or representing policemen^150–155^; BAT describing them as ‘the credible voice for contraband tobacco’.^156^

In 2016, PMI launched *PMI Impact* with $100 million to fund projects addressing illicit trade, corruption, organised crime and money laundering.^157^ With applications judged by an Expert Council of individuals closely linked to multiple UN agencies^158^ and Interpol,^159^ and PMI Impact’s September 2017 event featuring presentations from (and enabling PMI executives to link with), among others, WCO, OECD, Europol and numerous UN agencies including the UNODC (see second paragraph in this section), it would appear the initiative’s purpose is to further cement PMI’s access to authorities and undermine the WHO and the FCTC Secretariat among UN agencies. The first 32 recipients of funding (totalling approximately US$28 million) announced in September 2017^160^ include KPMG, Oxford Economics, Transcrime and others previously commissioned by PMI to produce widely criticised reports^20 25 161 162^ on illicit favourable to the TTCs.^159 161 162^

## Discussion

This evidence outlined in this paper indicates that the tobacco industry has created a T&T system it can control and, through an elaborate campaign involving front groups, third parties and increasingly complex relationships with other companies, all underpinned by a massive public relations effort, is aiming to have this system implemented as the global T&T system of choice under the ITP. Simultaneous evidence suggests it remains involved in tobacco smuggling. This combination of events would fundamentally undermine the ITP by enabling tobacco companies, with a vested interest in minimising their tax payments, to control the very system aimed to maximise those payments and reduce tobacco smuggling.

Three key findings underpin this conclusion. First, diverse and growing evidence shows that tobacco industry illicit outstrips the problems of cheap whites and counterfeits and remains the single largest problem in illicit tobacco; that incentives for industry involvement have barely changed since their well-documented involvement in the 1990s; that tobacco companies likely continue to be involved in and benefit from tobacco smuggling; and that this problem has persisted since the widespread introduction of Codentify. Possible interpretations are that Codentify is technically unfit for purpose or that TTC control renders Codentify useless.

Second, TTCs have a vested interest in controlling a T&T system intended to address tobacco industry illicit and fear T&T systems outside their control. This drove them to work collaboratively to oppose competitor systems, promote their own digital system, Codentify, and influence regulation on T&T to favour it. This collaborative campaign involved extensive subterfuge including the creation of front groups like DCTA to promote Codentify and channel funding to others who further promoted Codentify (eg, KPMG and WCO), and use of companies like FractureCode. Later elements of their intellectual property on Codentify were sold to other companies with Codentify now being promoted by companies and coalitions purporting to be independent yet having clear TTC links, including co-ownership of intellectual property rights to Codentify by former PM staff now at Inexto.

Third, underpinning all the above, was an extensive and well-funded stakeholder management and public relations effort involving funding for conferences, training, research, ex-policemen to act as spokespeople, and major organisations in the field including intergovernmental organisations. Such efforts are often operationalised via third parties (eg, DCTA, BASCAP) or specific initiatives (eg, PMI Impact). They serve to: cement the TTCs’ previously observed control over data and research on tobacco smuggling^6^; create and disseminate discourses favourable to industry; and build a network of influential organisations and individuals that support, promote and enhance the credibility of these misleading industry discourses. These efforts should be seen as part of a broader strategy to rehabilitate the TTCs’ image,^163–165^ reintegrate TTCs into policy-making circles from which they have been excluded, and undermine WHO and the Convention Secretariat among UN agencies. The concern that such efforts lead to regulatory capture is enhanced by findings from Africa that BAT has been paying to obtain data suggesting its competitors were smuggling and to gain influence with tax authorities.

### Limitations

Like any illegal activity, tobacco smuggling is complex, hidden and hard to investigate. We are limited to data that are publicly available and documents provided to us and cannot, therefore, access legal agreements between TTCs, DCTA and the various companies now promoting Codentify.

### Policy implications

The findings signal a very real danger of regulatory capture of the governmental and intergovernmental institutions responsible for addressing tax evasion and TTCs coming to control a global T&T system thereby fundamentally undermining it. Recent press reports from Argentina suggest these dangers may already be being realised with legal charges against PMI alleging its use of Codentify to hide levels of cigarette production in order to avoid paying taxes.^166^ By contrast we note the increase in legal tobacco sales and tobacco tax revenue in Kenya postimplementation of an independent T&T system (box 4).^167^

The findings indicate that determining independence from industry when operationalising the ITP is increasingly difficult. Experience in the EU^168^ suggests that definitions which require countries, possibly repeatedly, to search patent and trademark registers, investigate industry links and company sources of income, and so on, are best avoided.

We therefore suggest, broadly in line with Convention Secretariat recommendations,^169^ that governments should be alert to the likelihood that TTCs will continue to disguise their links to Codentify and that Codentify will be promoted under different names and by different companies. The safest response is for governments to assume that (1) the TTCs remain involved in any T&T system based on Codentify or on intellectual property currently or previously owned by a TTC and (2) such a system would be incompatible with the ITP and ineffective in reducing illicit trade within the legal supply chain. The industry’s own use of Codentify to help address counterfeiting should be seen as entirely separate. To help address potential regulatory capture, decisions on T&T should be cross-governmental and it is vital that health ministries are involved.

**What this paper adds**

**What is already known on this subject**

- The Framework Convention on Tobacco Control’s Illicit Trade Protocol (ITP) aims, inter alia, to secure the supply chain of legally manufactured tobacco products through a global track and trace (T&T) system. Given evidence of the tobacco industry’s (TI) historical involvement in cigarette smuggling, the protocol stipulates that such systems ‘shall not be performed by or delegated to the tobacco industry’. Philip Morris International developed a code-generating system, Codentify, and licensed it for free to its competitors in a deal which saw the four transnational tobacco companies agree to promote Codentify to governments as a T&T system.

**What this paper adds**

- Growing evidence indicates the TI remains involved in tobacco smuggling and therefore has a vested interest in controlling any T&T system aimed to control its supply in order to avoid scrutiny and minimise its excise payments.
- The TI’s attempts to have its Codentify-based system implemented as a T&T system have become increasingly underhand. They include claiming Codentify is independent of the TI by using front groups and front companies to promote it; selling some parts of its intellectual property on Codentify while retaining others, leaving a complex web of shared interests; paying networks of spies to obtain data showing its competitors are smuggling; and providing significant funding (administered directly and via third parties) for conferences, training, research and international police and anti-corruption organisations which serves to foment confusion over tobacco smuggling and create a powerful network supportive of the TI’s position.
- Governments should assume that any system based on Codentify, on intellectual property currently or previously owned by the TI, or being promoted by companies with TI links, is incompatible with the ITP and would not serve to reduce illicit trade within the legal supply chain.
