# Supplementary material for: Tobacco industry’s elaborate attempts to control a global track and trace system and fundamentally undermine the Illicit Trade Protocol
Source: Tob Control. 2018 Jun 13;28(2):127–40. doi: 10.1136/tobaccocontrol-2017-054191 (PMC6580790; doi:10.1136/tobaccocontrol-2017-054191)
Supplement: Supplementary file 1 [file tobaccocontrol-2017-054191supp001.pdf]

Appendix 1: Countries that have used Codentify	
Several sources associated with the distribution of Codentify claim that Codentify, in some variant, has been used in over 50 to 100 countries worldwide.[1-3] As these industry-linked sources do not detail the countries, these claims cannot be verified. Below is a list of countries which other named sources (identified below) suggest have implemented Codentify in some form.[4-6] We note, however, that this may simply signal the industry's internal use of Codentify as a product authentification tool rather than for track and tracing purposes and that equally, it may not signal government support for Codentify (see, for example[7])	
**Country**	**Source**
Belgium	4
Brazil	5
Bulgaria	4
Czech Republic	4
Denmark	4
Dominican Republic	6
Ecuador	6
Estonia	4
France	5
Germany	4 6
Greece	4
Guatemala	6
Hungary	4
India	5
Indonesia	5
Iraq	5
Ireland	4
Italy	4
Latvia	4
Lebanon	6
Lithuania	4
Luxemburg	4
Netherlands	4
Panama	5 6
Peru	6
Poland	4
Portugal	4 6
Romania	4
Russia	5
Slovakia	4
Spain	4
Switzerland	5
Turkey	5
UK	7
Ukraine	5

[1] Hubert D. *Implementing Technologies to Fight the Illicit Trade*. May 2013. Available:http://www.iticnet.org/file/document/watch/3686 (accessed 16 January 2018).
[2] Phelip B. *blue-infinity Traceability Workshop supports TPD II regulation readiness*. 29 April 2015. Available:https://www.blue-infinity.com/blog/2015/04/29/blue-infinity-traceability-workshop-supports-tpd-ii-regulation-readiness (accessed 16 January 2018).
[3] Lee B, Digital Coding and Tracking Association. *Effective Decision Making Empowered By Technology*. 23 Apr 2015. Available:http://iticnet.org/file/document/watch/4487 (accessed 03 Oct 2017).
[4] Action on Smoking and Health, Framework Convention Alliance, South East Asia Tobacco Control Alliance*, et al. Does the tobacco industry have a tracking and tracing system that governments can use?* 2015. Available:http://www.fctc.org/images/stories/Codentify_final_220515.pdf (accessed 16 January 2018).
[5] INTERPOL: Office of Legal Affairs. *COUNTERING ILLICIT TRADE IN TOBACCO PRODUCTS: A GUIDE FOR POLICYMAKERS*. Jun 2014. Available:https://www.interpol.int/News-and-media/Publications2/Guides-manuals/Countering-Illicit-Trade-in-Tobacco-Products-A-guide-for-Policy-Makers-June-2014 (accessed 06 Nov 2017).
[6] Campaign for Tobacco Free Kids, Colledge JW. *Tracing, Tracking & the Draft Illicit Trade Protocol*. February 2012. Available:http://www.tobaccocontrollaws.org/files/presentations/CTFK-ILC_Tracing_and_Tracking_Webinar_PPT_Presentation.pps (accessed 16 January 2018).
[7] Parliament.uk. *Tobacco: Excise Duties:Written question - HL6683*. 3 March 2016. Available:http://www.parliament.uk/business/publications/written-questions-answers-statements/written-question/Lords/2016-03-03/HL6683/ (accessed 16 January 2018).
