# Supplementary material for: Tobacco industry’s elaborate attempts to control a global track and trace system and fundamentally undermine the Illicit Trade Protocol
Source: Tob Control. 2018 Jun 13;28(2):127–40. doi: 10.1136/tobaccocontrol-2017-054191 (PMC6580790; doi:10.1136/tobaccocontrol-2017-054191)
Supplement: Supplementary file 2 [file tobaccocontrol-2017-054191supp002.pdf]

APPENDIX 2

Figure 1. Screengrabs: World Intellectual Property Organization (WIPO) Database search for "Codentify" via www.TMview.org, showing change of ownership for CH (Swiss) and WO trademarks

Figure 1a: 13 October 2016 – PM listed as Applicant

Advanced search | Filters

st of results

WIPO Global Brand DB — Page 1 of 1 — 10 20 30 50 75 100 | Displaying results 1 -

	Graphic repres...	Trade mark name	Trade ...	Desig...	Application ...	Trade mark ...	Nice class	Applicant name	Application ...	Trade mark type	Registratio...	Seniori
	-	CODENTIFY	CH	CH	56378/2006 548516	Registered	9,42	Philip Morris Products S.A.	19-07-2006	Word	21-07-2006	
+	-	CODENTIFY	WO	JP, LV, LU, BY, LT, VN, GB, HR, RO, TR, DZ, NO, HU, FR, BG, ME, MK, BE, DE, DK, FI, MA, IE, AT, CZ, CY, SE, KR, SI, SK, IT, BA, MT, PT, PL, RS, UA, EM, RU, GR, ES, NL, EG, EE, CN	917422 917422	Registered	9,42	Philip Morris Products S.A.	17-01-2007	Word	17-01-2007	
	-	CODENTIFY	MX	MX	0826417 982271	Registered	9	PHILIP MORRIS PRODUCT...	19-12-2006	Word	25-04-2007	
	-	CODENTIFY	MX	MX	0826418 973655	Registered	42	PHILIP MORRIS PRODUCT...	19-12-2006	Word	22-02-2007	

Figure 1b: 11August 2017 – Inexto now listed as the Applicant on the CH and WO trademarks (albeit with reduced number of countries now listed under WO)

List of results

WIPO Global Brand DB — Page 1 of 2 — 10 20 30 50 75 100 | Displaying results 1 - 10 of 12

	Graphic represent...	Trade mark name	Trade m...	Designa...	Application nu...	Registration n...	Trade mark st...	Nice class	Applicant name	Application date	Trade mark type	Registration d...	Seniority clai...
	-	CODENTIFY	MX	MX	0826417	982271	Registered	9	PHILIP MORRIS PRODUCTS S.A.	19-12-2006	Word	25-04-2007	
	-	CODENTIFY	MX	MX	0826418	973655	Registered	42	PHILIP MORRIS PRODUCTS S.A.	19-12-2006	Word	22-02-2007	
	-	CODENTIFY	BR	BR	828924066	828924066	Registered	9	PHILIP MORRIS PRODUCTS S.A.	29-12-2006	Word	16-03-2010	
	-	CODENTIFY	BR	BR	828924074	828924074	Registered	42	PHILIP MORRIS PRODUCTS S.A.	29-12-2006	Word	16-03-2010	
	CODENTIFY	CODENTIFY	US	US	86964399	5061134	Registered	9	Philip Morris USA Inc.	05-04-2016	Word	11-10-2016	
	CODENTIFY	CODENTIFY	PH	PH	PH-4-2006-13890	4-2006-13890	Ended	9,42	PHILIP MORRIS PRODUCTS S.A.	22-12-2006	Combined	30-07-2007	
	-	CODENTIFY	CH	CH	56378/2006	P-548516	Registered	9,42	Inexto SA	19-07-2006	Word	21-07-2006	
	CODENTIFY	CODENTIFY	MY	MY	07000341	07000341	Registered	42	PHILIP MORRIS PRODUCTS S.A.	09-01-2007	Stylized characters	30-10-2008	
	CODENTIFY	CODENTIFY	MY	MY	07000340	07000340	Registered	9	PHILIP MORRIS PRODUCTS S.A.	09-01-2007	Stylized characters	08-09-2008	
+	-	CODENTIFY	WO	LV, LU, LT, GB, HR, RO, HU, BG, FR, BE, DE, DK, FI, IE, CZ, AT, CY, SE, SI, SK, IT, MT, PL, PT, EM, GR, ES, NL, EE	917422	917422	Registered	9,42	INEXTO SA	17-01-2007	Word	17-01-2007	

View/Hide columns — Reset columns — Remember columns — Page 1 of 2 — 10 20 30 50 75 100 | Displaying results 1 - 10 of 12
